# Enhancement of oil productivity of *Mortierella alpine* and investigation into the potential of Pickering oil‐in‐water emulsions to improve its oxidative stability

**DOI:** 10.1002/fsn3.2651

**Published:** 2021-11-02

**Authors:** Marjan Esfandiyari Mehni, Hamid Reza Samadlouie, Ahmad Rajaei

**Affiliations:** ^1^ Department of Food Science and Technology Faculty of Agriculture Shahrood University of Technology Shahrood Iran

**Keywords:** chitosan, *Mortierella alpine*, oil, oxidative stability, pickering emulsion

## Abstract

*Mortierella alpine* is an oleaginous fungi known for its tendency to produce oil and polyunsaturated fatty acid. Initial experiment indicated that magnesium oxide nanoparticles (MgONPs) accelerated glucose consumption and, consequently, oil production. After enhancement of *Mortierella alpine* CBS 754.68' oil production, the oxidative stability of the oil rich in long‐chain polyunsaturated fatty acids (arachidonic acid) encapsulated by modified chitosan (CS) was assayed. To confirm the modification of CS, Fourier transform infrared spectroscopy (FTIR) spectrum indicated that the connection between CS and capric acid (CA) as well as stearic acid (SA) was well formed, leading to a considerable improvement in nanoparticle formation, measured by the SEM photographs, and physical and oxidative stability of emulsions. The oxidative stability of *Mortierella alpine'* oil emulsion in a period of 20 days at ambient temperature was monitored. Of all treated media, CS‐SA nanoparticles were of the most oxidative stability. The rheological tests showed that viscosity behaviors were dominated by elastic behaviors in the impregnating emulsion with unmodified CS at the applied frequencies, and the elastic behavior of the emulsion sample prepared with CS‐SA was slightly higher than that of the emulsion prepared with CS‐CA. The results of redispersibility indicated that the powdered emulsion stabilized by CS‐SA had the lowest water absorption.

## Graphical abstract

The oxidative stability of Mortierella alpine oil encapsulated by modified chitosan was investigated. CS‐stearic acid nanoparticles were of the most oxidative stability.

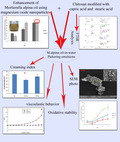

## 1 INTRODUCTION

Oil‐in‐water (O/W) emulsions as colloidal systems are of a great potential for a number of commercial and industrial applications (Song et al., 2015). Surely, **e**ncapsulations of the nutraceuticals to maintain protection of the oil functionality from the oxidative stress driven by environmental stresses have been recognized as the main applications of the emulsions for commercial exploitations (de Vos et al., 2010). Considering the fact that the high free energy contributing to the interfacial tension at the Oil/Water interface causes the physical and chemical instability, which subsequently results in oil oxidation and destabilization of the emulsion structures (McClements & Decker, 2000). To reduce this destructive surface energy, various scientific approaches have been proposed. Of all methods scientifically approached, particle‐stabilized emulsions, named Pickering emulsions, play a vital role in stabilizing emulsion droplets against aggregation and oxidation (Nicolai & Murray, 2017; Wang et al., 2021). To be more precise, polymeric steric stabilization as short‐range repulsive interaction generated by these kinds of emulsifiers is efficacious in stabilizing emulsions (Ekaterina & Oleg, 1991). Correspondingly, the manipulation of the thickness of interfacial layer by using various stabilizing particles such as cellulose, proteins, fat crystals, and chitin nanocrystals has been identified as a major contributing factor in generating a strong steric repulsion and, consequently, emulsion stabilization (Errezma et al., 2018; Song et al., 2015). Chitosan (CS) as a basic linear polysaccharide has been found to capture the attention of scientists who want to determine the potential use of CS as a emulsifier to stabilize Pickering emulsions (Olawuyi et al., 2019; Shah et al., 2016). On the contrary, high hydrophilic property is one of the most frequently stated problems with CS‐stabilized Pickering emulsion (He et al., 2017; Nan et al., 2014). Given that, the discovery of the surface modifications of CS to improve the hydrophobic properties triggered a huge amount of innovative scientific inquiry (Hosseini et al., 2019). CS particles have been modified with various components such as gallic acid (Guo et al., 2016), monosaccharides (Il'ina et al., 2008), and fatty acids (Mirzaee Moghaddam & Rajaie, 2021), imparting some desired functional properties (Elsabee et al., 2009; Ou et al., 2015). Among various practical applications of Pickering emulsions in last few decades, there has been a surge of interest in the protective effect of Pickering particles on oil droplets (Li et al., 2021). Among various edible oils, *Mortierella alpine'* oil has been attracted a significant attention of scientists. *Mortierella alpine* as a commercial source of arachidonic acid can accumulate substantial amounts of oil in its biomass (Goyzueta‐Mamani et al., 2021; Wynn & Ratledge, 2000). Notably, carbon and protein sources as key substrates give high impacts on oil and arachidonic acid productions (Samadlouie et al., 2018). Among various key macroelements, magnesium (Mg2+) as a cofactor of different enzymes stimulates a net rise in oil accumulation (Sissi & Palumbo, 2009). Recent research indicated that nanoparticles are able to enhance the microbiological reaction rates (Gonabadi et al., 2021; Shan et al., 2005). Therefore, after the optimization of chemical conditions for oil production by *Mortierella alpine CBS 754.68*, the present study was first attempt made to protect *Mortierella alpine'* oil using a Pickering emulsion. Initially, CS was modified, and subsequently, its emulsifying properties were analyzed. Finally, the oxidative stabilities of the Pickering emulsions were investigated.

## 2 MATERIALS AND METHODS

### 2.1 Materials

CS (50–190 KDa) was purchased from Sigma–Aldrich. Methanol, acetic acid, NaOH, Tween 80, SA, ethanol, ammonium thiocyanate, iron (II), trichloroacetic acid, and cumene hydroperoxide were purchased from Merck. Chloroform was obtained from Samchun. 1‐Ethyl‐3‐(3‐dimethylaminopropyl) carbodiimide (EDC) was purchased from Fluka. All materials were used without further purification.

### 2.2 Fermentation

*Mortierella alpine* CBS 754.68 was purchased from Central bureau Schimmelcultures (CBS, the Netherlands). The strain was incubated in medium containing 30 g/L glucose and 7 g/L yeast extract as seed culture. The 10% (v/v) of the seed culture was added to fermentation medium then incubated in shack flasks at 21°C, pH 6 for 5 days. To induce oleaginous potential, nitrogen‐limited media (10 g/L yeast extract) with relatively high glucose content (70 g/L) were designed (Samadlouie et al., 2012). MgONPs were also used to assess its effect on *Mortierella alpine* CBS 754.68 metabolisms. MgONPs were attained from US Research Nanomaterials Inc., which had 99% purity and an average size of 20 nm with polyhedral morphology.

### 2.3 Determination of substrates consumption and metabolic productions

The biomass and fermentation broth were separated by vacuum filtration via Whatman No. 4 filter paper. No.1 Whatman paper was served to collect biomass washed with water twice, then freeze‐dried to evaluate dry weight biomass (DWB) and oil content according to the method of (Jang et al., 2005). Fermentation broth was used for the analysis of residual reducing sugar and nitrogen content. Reducing sugar concentration was assayed by using dinitrosalicylic (DNS) colorimetric method (Miller, 1959). The Lowry's method was served to determine nitrogen concentration (Lowry et al., 1951).

### 2.4 Fatty acid methyl ester evaluation

To obtain fatty acid methyl esters, 5 mL of methanolic NaOH (0.5 N) and 1 mL hexane were added to 0.1 g of *Mortierella alpine'* oil in a screw‐cap vial. Thereby, the mixture was heated in boiling water for 10 min. After cooling at ambient temperature, 2.175 mL of methanolic boron trifluoride (BF3) was added to the solution and heated in boiling water bath for 3 min. After cooling, 1 mL saturated sodium chloride and 1 mL hexane were added to the mixture. The mixture was shaken thoroughly. After 5 min, 0.2 µL of the top hexane layer‐containing methyl ester was removed to inject onto the head of gas chromatograph (GC; Unicam 4600) and then was analyzed by (Metcalfe et al., 1966) method. A fused silica capillary column (BPX70; SGE) which had 30 m × 0.25 mm × 0.22 m film thickness, and a split injector (1.2 L injections) at 250°C and FID at 300°C was used to determine the fatty acid profiles of *Mortierella alpine'* oil. Helium was used as the carrier gas (pressure of 20 Psi). The temperature of the column was 190°C. The peaks were identified according to their retention times using fatty acids methyl esters standards. All samples were run in triplicate. An internal standard method was used to calculate the fatty acid composition (the internal standard was C15:0) (Metcalfe et al., 1966).

### 2.5 Preparation and characterization of CS‐CA and CS‐SA

For preparing CS‐CA and CS‐SA, the amide bonds were formed between the carboxylic acid groups of SA and CA with the free amino groups of CS via an EDC‐mediated reaction using the method described by Hosseini & Rajaei, (2020) with minor modification. In this study, CA‐to‐CS and SA‐to‐CS ratios were 0.5–1.

To confirm the formation of the amide bonds between CS chains with CA and SA, Fourier transformation infrared (FTIR) spectrum at 20°C and at the range of 500–4,000 cm^−1^ was achieved using an FTIR‐430 (Jascow). The degree of deacetylation (DD = 82.7%) of CS was determined by the IR spectroscopy method (Equation 1) (Sabnis & Block, 1997). Thereby, the degree of amidation (DA) of nanogels was identified by FTIR spectrums using the following equation (Equation 2) (Sabnis & Block, 1997).

(1) $$\text{DD}=97.67‐[26.486‐\left(A_{1634}/A_{3430}\right)]$$

(2) DA=100‐DD

In addition, SEM (Philips XL30, FEI, Hillsboro, OR) was applied to assess the morphology of the CS‐SA and the CS‐SA particles.

### 2.6 Preparation of *Mortierella alpine* oil‐in‐water Pickering emulsions

*Mortierella alpine* oil‐in‐water Pickering emulsions were prepared according to our previous method (Atarian et al., 2019). To investigate the effect of type of stabilizer (CS, CS‐CA, and CS‐SA) on the stability of emulsion, Pickering emulsions were prepared at a fixed 20% Mortierella oil ratio, 2% CS‐SA stabilizer, and pH 8. After preparation of emulsions, the samples were transferred into glass containers and then kept at ambient temperature. In order to confirm the emulsion type, the drop test was used (Feng & Lee, 2016).

### 2.7 Creaming stability

Creaming index (CI) was served to determine the creaming stability of the emulsions. To calculate CI index, after 2 weeks, method of (Cheong et al., 2016) was used according to the following equation:

(3) $$\text{CI (}\%)=(H_{s}{\text{/H}}_{t})\times 100$$

where H_s_ is the height of the serum layer and H_t_ is the total height of the formulation (H_t_) after 2 weeks of storage at ambient temperature.

### 2.8 Microstructure of emulsions

GX optical microscope with a CCD camera (CM TCAM3) was served to microscopically analyze the droplet size of Pickering emulsions after 3 h of storage. The mean droplet diameter was determined using Image J1.46 software. To assure exact particle size, nine images were taken from each sample.

### 2.9 Rheological Measurements

A rheometer (Modular Compact Rheometer) was used to evaluate the flow characteristics of the Pickering emulsions stabilized by CS, CS‐CA, and CS‐SA. The viscoelastic properties of the emulsions were measured by cone and plate geometry (1° cone angle, 50 mm diameter, and 54 µm gap). A rest time of 600 s was selected for all rheological tests. The linear viscoelastic range was measured with a strain sweep (0.01%–100%) at a fixed frequency of 10 rad/s. Dynamic tests were performed in the linear viscoelastic region (strain amplitude of 0.5%), and the storage modulus (G') and loss modulus (G") against frequency were plotted. Frequency scanning test was performed at 20°C (Hosseini & Rajaei, 2020).

### 2.10 Assessment of oil oxidation

To evaluate the oxidative stability, *Mortierella alpine* oil‐in‐water Pickering emulsions prepared by different stabilizer (CS, CS‐CA, and CS‐SA particles) were placed at 35°C for 20 days and the production of hydroperoxides was evaluated (Hosseini et al., 2019) at different time intervals. Briefly, 10 µL of each sample was first added to 8.9 mL of chloroform/methanol (3:7 v/v) and mixed for 2–4 s. Then, 50 µl of 30% ammonium thiocyanate (w/v) solution was added and stirred for 2–4 s; after that, 50 µl of ferric chloride II solution was added to the samples. After 20 min, the absorbance of the solutions was read at 500 nm using a UV‐Vis spectrophotometer (UV2150 model). To obtain the standard curve, the concentrations of 0.5–3 ppm of cumene hydroperoxide were used. The peroxide value (PV) was expressed as mmol hydroperoxides in kg of oil (Salminen et al., 2014).

### 2.11 Redispersibility assay

To measure the redispersibility of different emulsions, first a certain amount of different emulsions stabilized with different stabilizers were dried by a freeze dryer (Alpha 1–2 LDplus, Christ). Then, a thin layer with a diameter of 3 mm was prepared from each of the powdered samples. A drop of 2 µL of distilled water was placed on the surface of the samples. Finally, the drop was photographed at different times using a contact angle measuring device (DCAM model, Adecco) (Ji et al., 2016).

### 2.12 Statistical analysis

All the experiments were done in triplicates unless otherwise is stated. The means and standard deviations (SD) were calculated using Microsoft Excel (2013) software. The SPSS 16.0 software was used to compare the differences among means at the level of 0.05 using Duncan's multiple range test.

## 3 RESULT AND DISCUSSION

### 3.1 Glucose and protein consumption during fermentation

**FIGURE 1:**
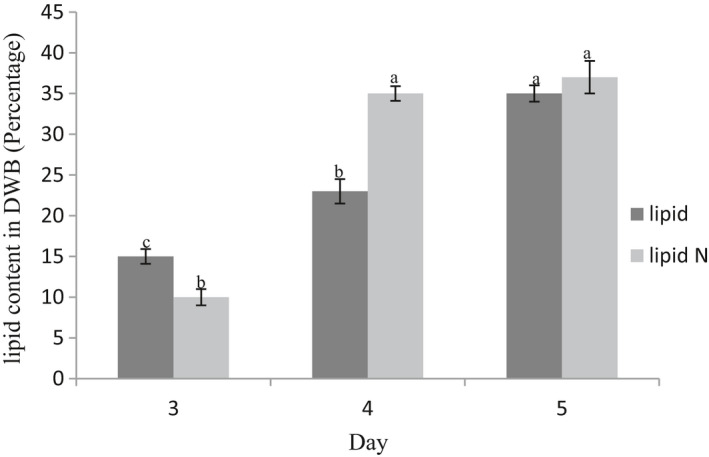
Percentage of oil (medium without MgONPs) and oil N (medium with MgONPs) accumulation in DWB over the 5‐day fermentation

The initial examination indicated that 0.005 g/L was an optimal level of MgONPs to stimulate *Mortierella alpine CBS 754.68* metabolites. On the other hand, the trend was reversed as MgONPs concentration increased (data not shown). Thereby, a 0.005 g/L MgONPs was used in the medium containing glucose and yeast extract. Oil production and glucose consumption were monitored simultaneously during fermentation. The results indicated that the protein substrate was used up completely on first day (data not shown); following that under starvation condition, the excess quantity of carbon remaining in medium was assimilated by fungi and converted into oil. Recent researches asserted that various carbon sources like rice bran, wheat bran, potato waste, molasses, potato starch, and rice straw were suitable for oil and biomass stimulations (Jang et al., 2005; Samadlouie et al., 2018). More to the point, the uptake of glucose by oleaginous microorganism is far easier than that of the other carbon sources. Glucose was claimed to be by far the best substrate for growth and oil production by oleaginous fungi (Hao et al., 2015). The data showed that the rate of glucose consumption was the highest between 3th and 4th day in medium without MgONPs. The rate of glucose consumption varied significantly over the 5 days of fermentation in the medium supplemented with MgONPs. In the first 3 days, the trend of glucose consumption was slower than two last days. To be more precise, the rate of glucose consumption was the highest between 3th and 4th day. (Figure 1).

### 3.2 Oil production during the fermentation time

As can be seen in Figure 1, in the medium without MgONPs, oil content experienced a continual rise during the whole of fermentation time. However, such upward trend was the least between 3th and 4th day. Notably, oil accumulation considerably promoted once glucose came to the least amount of it. It is postulated that the absorbed glucose might have been converted into oil. This result was in agreement with (Hwang et al., 2005), having been reported considerable amount of oil was accumulated even when the glucose content dipped as low as 10 g/L. The result indicated that the upward trend of oil production varied during the fermentation in medium containing MgONPs. The highest amount of oil was accumulated between 3th and 4th of fermentation (Figure 1). All in all, oil of *Mortierella alpine* dry biomass at 5 days in medium supplemented with MgONPs was extracted and used for further processes such as fatty acid analysis, Pickering emulsion preparation, and determination of the emulsion properties.

### 3.3 Fatty acid profile of *Mortierella alpine* oil

**FIGURE 2:**
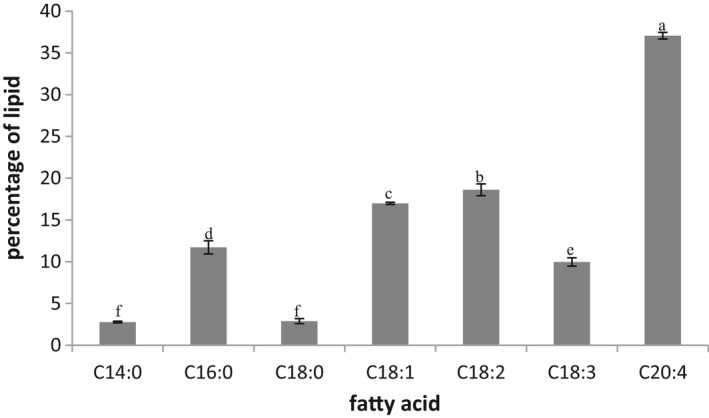
Fatty acid profile (percentage of oil) of *Mortierella alpine oil*

As can be seen in Figure 2, unsaturated fatty acids comprised some 68.42 percent of total fatty acid content, and importantly, more than half of it was made of arachidonic acid. With Mortierella alpine having high content of polyunsaturated fatty acid, the oil appeared quite sensitive to the oxidative stress.

### 3.4 Characteristics of CS‐CA and CS‐SA

**FIGURE 3:**
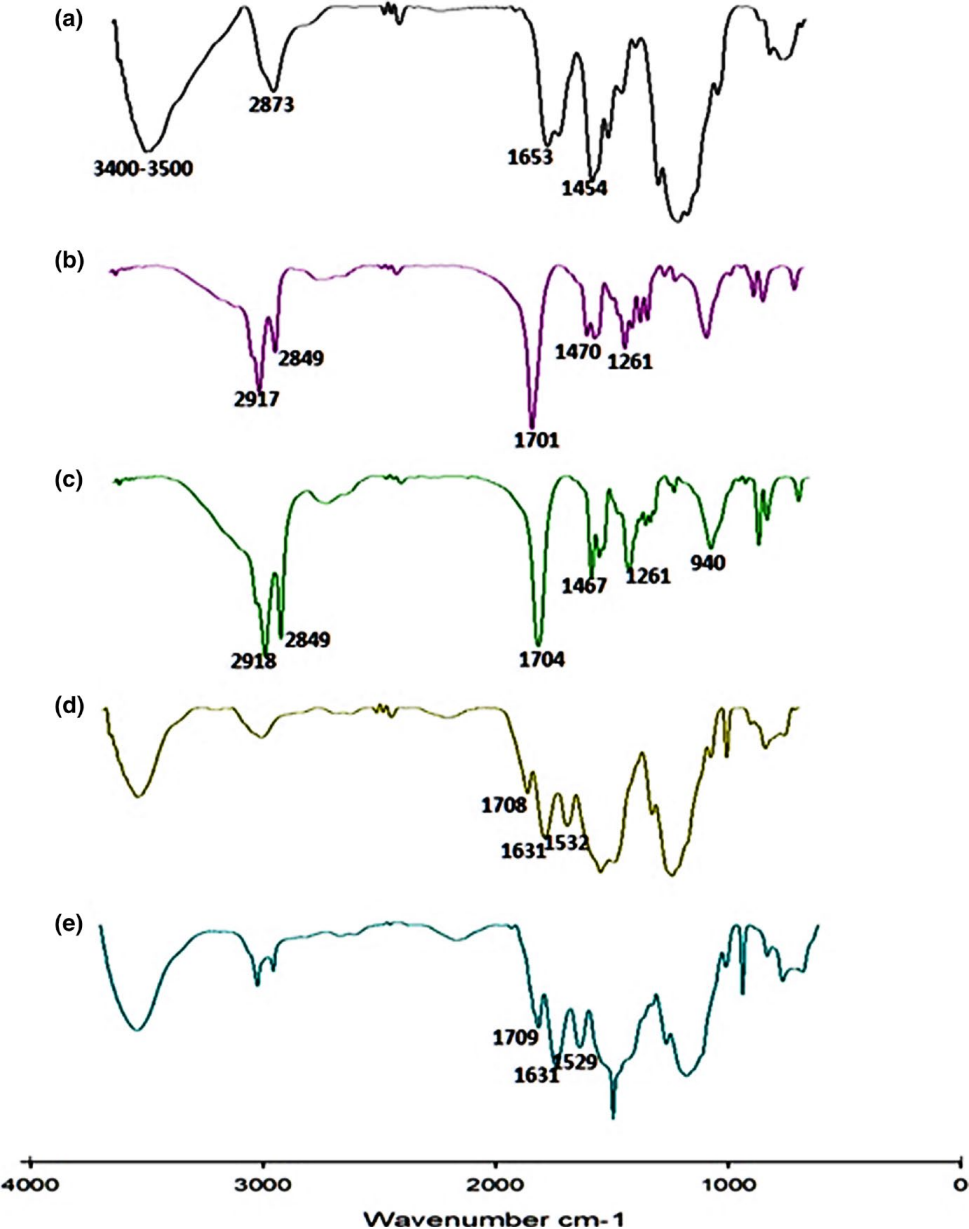
Fourier transform infrared spectroscopy (FTIR) analysis obtained for CS (a), CA (b), SA (c), CS‐CA (d), and CS‐SA (e)

**FIGURE 4:**
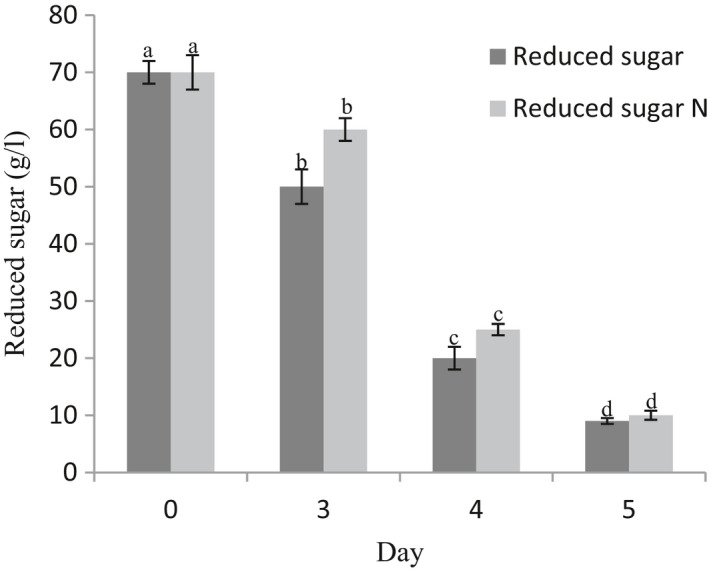
Reduced sugar (medium without MgONPs) and reduced sugar N (medium with MgONPs) over the 5‐day fermentation

**FIGURE 5:**
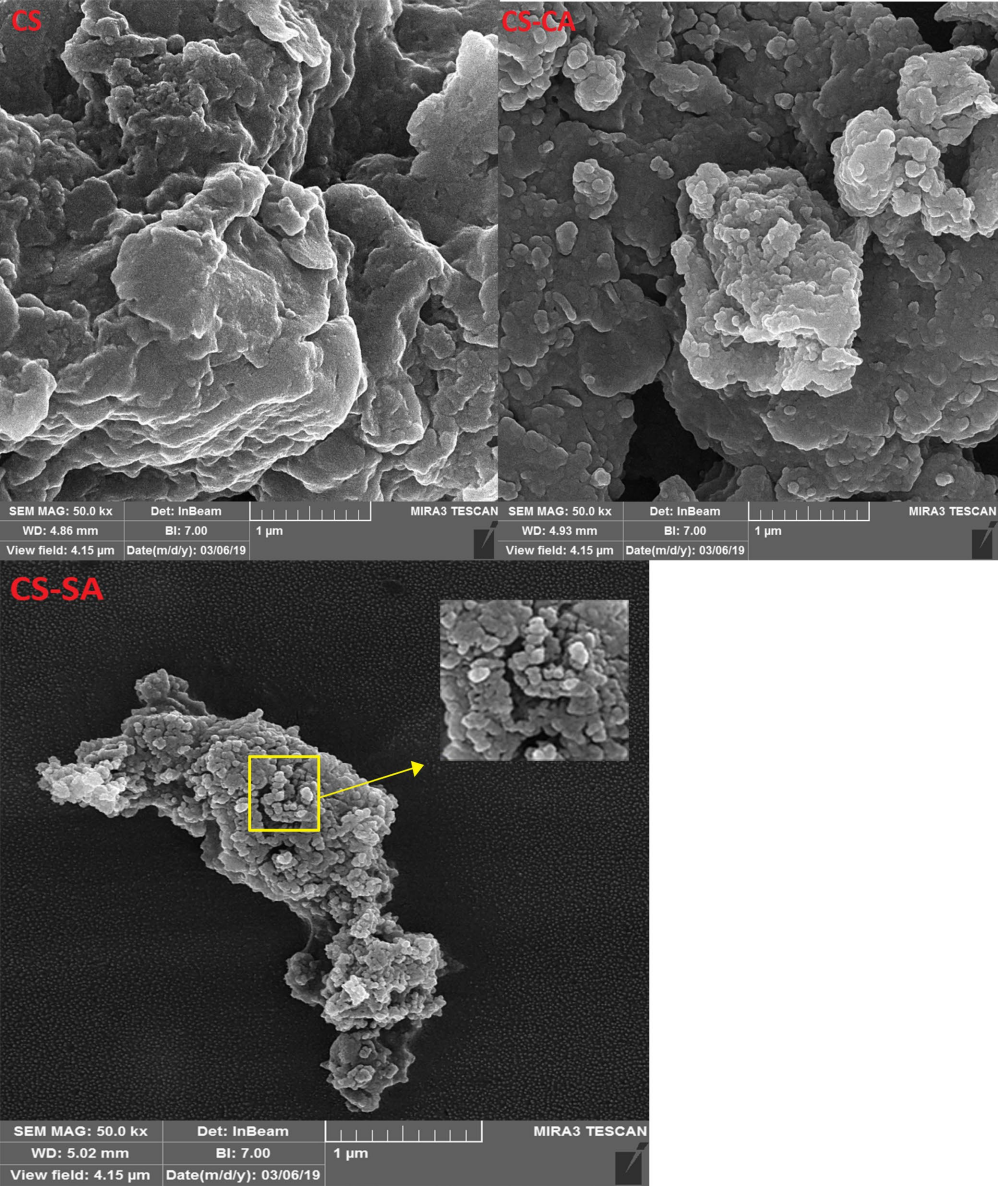
Scanning electron microscopy (SEM) micrographs of CS, CS‐CA, and CS‐SA particles

Covalent cross‐linking was formed between the free amino groups of CS and the carboxylic acid groups of CA and SA to improve hydrophobic property of CS. The structural characterizations obtained by FTIR spectroscopy for CS, CA, SA, CS‐CA, and CS‐SA were shown in Figure 3. As can be seen in Figure 3a, absorption peak at 3,400–3,500 cm^−1^ related to the overlay of the −OH and −NH_2_ groups. The peak placed at 2,873 cm^−1^ provided an evidence of the stretching vibration of CH_2_. In addition, the peak at 1,653 cm^−1^ was related to the C = O stretching vibrations (Atarian et al., 2019). In Figure 3b, related to CA, the stretching vibration of C‐H of CH_2_ groups observed at 2,917 and 2,849 cm^−1^; respectively, the peak at 1,701 cm^−1^ provided evidence of the presence of the stretching vibration of C = O on carboxylic fatty acid group. The peak at 1,470 cm^−1^ corresponded to the bending vibration of C‐H on CH_2_ and CH_3_ group of fatty acids. Absorption peaks at 1,260 cm^−1^ were related to the presence of stretching vibration of C‐O on carboxylic fatty acid (Larkin, 2011). Figure 3c shows the SA spectrum. The peaks observed in the SA spectrum are approximately similar to the peaks in Figure 3b. The spectrum of CS‐CA was represented in Figure 3d. C = O and N‐H bonds of the amide group created peaks at 1,708, 1,631, and 1,532 cm^−1^, respectively. These observed peaks were due to interaction of CS with the CA which revealed that the amide bonds between CS and CA were well formed. The same went for CS‐SA Figure 4e. 1,709, 1,631, and 1,529 peaks in spectrum related to C = O and N‐H bonds of the amide group, respectively, supported the claim that the amide bonds between CS and SA are well formed. In addition, the DA of CS‐CA and CS‐SA nanogels was 28.3 and 28.2%, respectively. The SEM micrographs for CS, CS‐CA, and CS‐SA particles were presented in Figure 5. As can be seen in Figure 5, it could be stated that the CS‐SA particles were more uniform and spherical in shape than CS‐CA; by contrast, CS had no uniform shape. The spherical particles of CS‐CA and CS‐SA indicated that well structures of particle had been formed as CS was modified by CA and SA. The hydrophobicity of the CA and SA exerted a great influence on the physical and chemical property of particles. According to Figure, CS‐SA particles were better off than that of CS‐CA particles. As can be seen in Figure 5, the structure indicated that CS nanoparticles were not well formed, while in the case of CS‐CA and CS‐SA particles, spherical particles have been created in the nanoscale. As a result, addition of CA and SA‐to‐CS chains made a considerable improvement in particle formation. Hydrophobicity of CA and SA had a positive impact on nanoparticle formations. These results were consistent with previous findings (Atarian et al., 2019; Hosseini et al., 2019; Shahbazi et al., 2020). It can be seen in Figure 5 that the structure of nanoparticles in CS‐SA was better than CS‐CA due to more hydrophobicity of its longer length of SA compared with CA.

### 3.5 Characteristics of *Mortierella alpine* oil‐in‐water Pickering emulsions

#### 3.5.1 Creaming index

**FIGURE 6:**
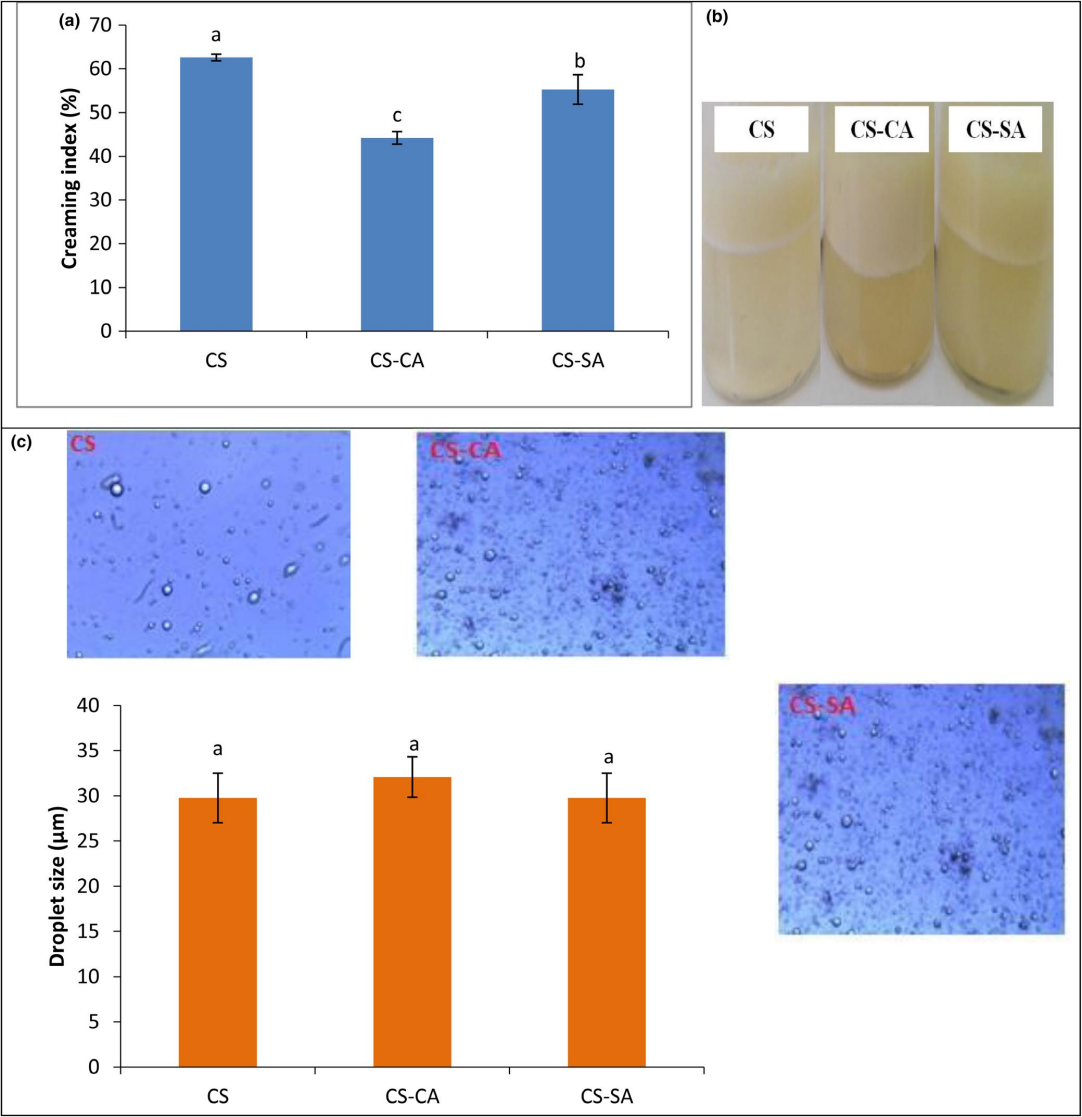
Creaming index (a), appearance after 14 days of storage (b), and droplet size (x) of Mortierella oil‐in‐water Pickering emulsions stabilized by CS, CS‐CA, and CS‐SA

As can be seen in Figure 6a,b, the creaming index of emulsion stabilized by CS was higher than that of emulsions stabilized by CS‐CA and CS‐SA. The greatest physical stability of modified CS (CS‐CA and CS‐SA) indicated that the fatty acids (CA and SA) attached to the CS chains improved the hydrophobicity of the CS. Previous findings postulated that CS modification enhanced the CS stability through binding between the amino acid groups in CS and the carboxylic groups of fatty acids (Atarian et al., 2019; Elsabee et al., 2009; Hosseini et al., 2019; Shahbazi et al., 2020). The droplet size of different *Mortierella alpine* oil‐in‐water Pickering emulsions after 3 h of storage was shown in Figure 6c. To calculate numerically the oil droplet size, the microscopy image and image J software were applied, indicating that emulsion stabilized by CS‐CA with 32.08 µm had the largest size after 3 h, while the smallest droplet size, namely 28.95 µm, was attained in emulsion stabilized by CS‐SA.

#### 3.5.2 Viscoelastic properties of Pickering emulsions

**FIGURE 7:**
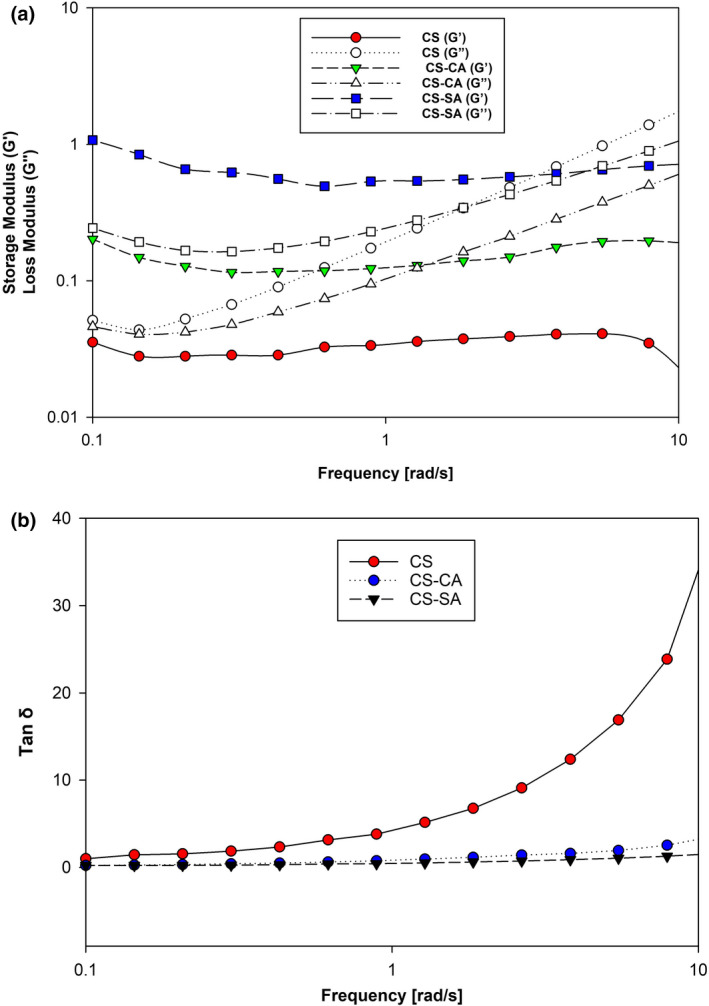
Dynamic frequency sweep (a) and tan δ (b) of O/W emulsions stabilized by CS, CS‐CA, and CS‐SA particles

Dynamic frequency sweep was performed to evaluate the viscoelastic behavior of different emulsions. The results of the oscillation tests were shown in Figure 7. The viscous behavior was superior to the elastic behavior in the Pickering emulsion with unmodified CS (Figure 7a). This result shows that the emulsion prepared with CS at a concentration of 20% oil was liquid. In the case of CS‐SA‐stabilized emulsion, from frequency 0.1 to about 6, the elastic behavior was higher than the viscous behavior, but at frequencies higher than 6, the viscous behavior was more dominant than the elastic behavior. In the case of the emulsion prepared with CS‐CA from frequency 0.1–1, the elastic behavior prevailed over the viscous behavior, and in the case of the emulsion prepared with CS‐SA, the viscous behavior prevailed at higher frequencies. The results of the tan δ for different Pickering emulsions were shown in Figure 7b. Tan *δ* provides information about the nature of the emulsion system's viscoelastic response: if the tan *δ* value approaches 1, then the emulsion is liquid‐like/viscous and if the tan *δ* value is less than 1, and the material has solid‐like/elastic behavior (Miao et al., 2015). According to Figure 7b, for both CS‐CA and CS‐SA stabilized emulsions, the tan *δ* value of <1 was observed in low frequencies, suggesting a solid‐like behavior of emulsion, but the tan *δ* value of >1 was observed in all frequencies for CS‐stabilized emulsion, suggesting a liquid‐like behavior. These results show that emulsions prepared with modified CS were solid‐like at low frequencies and liquid‐like at high frequencies. Frequency dependent was also observed in the case of emulsions prepared with modified CS, which indicates that a gel network was formed in the emulsion structure. This result was consistent with the work of other researchers. Including a study on an emulsion‐stabilized Pickling emulsion with insoluble starch nanoparticles (ISNP) and actinyl‐soluble starch nanoparticles (OSA‐SSNP), the results showed that G' was always greater than G' that both modules depended frequency on a whole frequency range. This result showed the presence of a gel‐like structure in the O/W emulsion system, which was a frequency dependence on a common behavior of weak gels (Miao et al., 2015). Also, according to Figure 7, it can be seen that the elastic behavior of the sample of emulsion prepared with CS‐SA was slightly higher than the emulsion prepared with CS‐CA. This result indicated that the gel matrix formed in the CS‐SA‐stabilized emulsion was slightly stronger than the CS‐CA‐stabilized emulsion. Due to the difference between SA and CA in terms of hydrocarbon chain length, it can be concluded that modifying the structure of CS with longer chain acids could lead to the productions of emulsion with more gel properties.

#### 3.5.3 Measurement of oxidative stability

**FIGURE 8:**
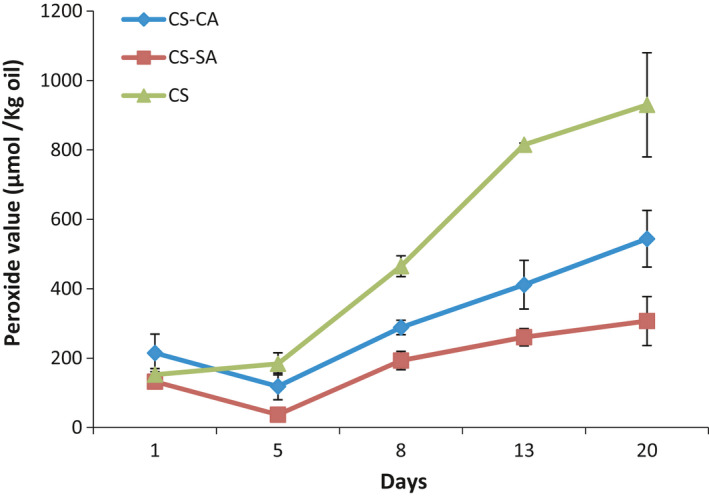
Peroxide values of Pickering emulsions stabilized by CS, CS‐CA, and CS‐SA

The oxidative stability of the chitosan, chitosan‐stearic acid nanogel, and chitosan‐capric acid nanogel emulsions was assayed by measuring the amount of peroxide contents of three different samples on days 1, 5, 8, 13, and 20 at ambient temperature (Figure 8). The results showed that the peroxide content ranged between 150 and 200 µmol/kg oil on the first day. The susceptibility of *Mortierella alpine* oil to oxidation along with vigorous shaking and exposure to ultrasound waves used would be considered as the main factors affecting oil deterioration at that time. Peroxide contents of all samples experienced upward trends during the examined days. There were vast differences in peroxide contents at all treatments such that the CS‐SA‐stabilized emulsion had the highest stability and the CS‐stabilized emulsion had the lowest oxidative stability. Recent research indicated that there are two main approaches for oxidative stabilization of emulsion by polymer emulsifiers, including increasing emulsion viscosity and steric repulsion of thickened polymer, covering the surface of the droplets (McClements & Decker, 2000). Latter seems to be regarded as a main factor in stabilizing the Pickering emulsion. According to SEM images (2), low affinity of CS chains to bond on the surface of the oil droplets and to form nanoparticles due to the high hydrophilicity of CS filaments (Elsabee et al., 2009) could be regarded as a main factor to increase the viscosity of the continuous phase. Such modifications increase hydrophobicity of the modified CS, leading to complete oil surface coverage and subsequently considerable enhancement in steric repulsion power. Such efficient polymeric coverage acts like barrier and protects the vulnerable emulsion of the *Mortierella alpine'* oil from the peroxidants present in continuous phase. According to Figure 8, it can be seen that the oxidative stability of CS‐SA‐stabilized emulsion was higher than that of CS‐CA‐stabilized emulsion. The highest oxidative stability of CS‐SA‐stabilized emulsion could be related to the highest hydrophobicity of CS‐SA particles. These capabilities of Pickering emulsion have been well established from a variety of researches (Atarian et al., 2019; Kargar et al., 2011, 2012).

#### 3.5.4 Measurement of redispersibility of powdered Pickering emulsions

**FIGURE 9:**
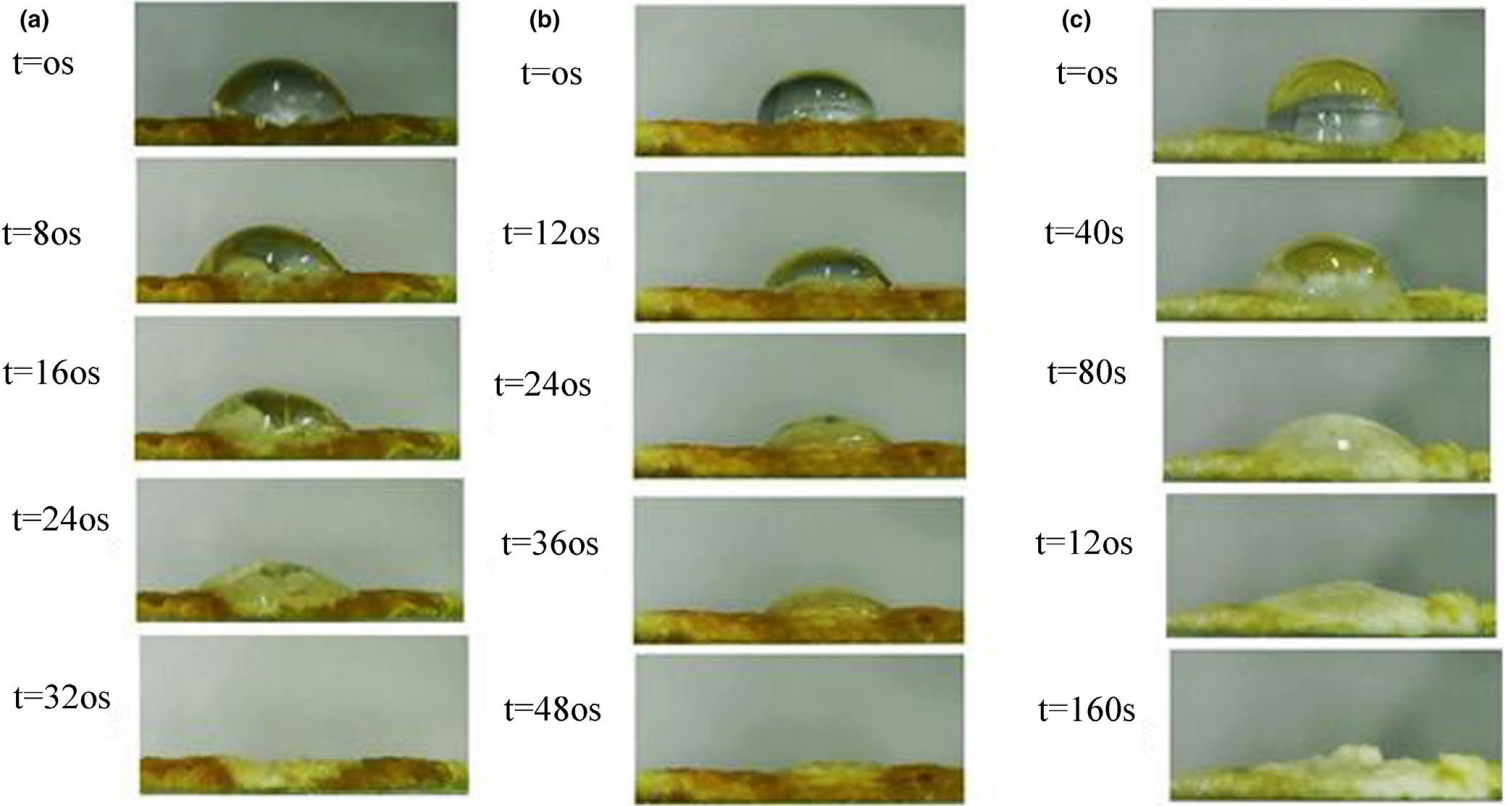
Redispersibility of powdered emulsions (a) stabilized by Cs, (b) stabilized by CS‐SA, and (c) stabilized by CS‐CA

As can be seen in Figure 9, CS‐stabilized emulsion powder was able to absorb water faster than others. High hydrophobicity of modified CS with SA caused emulsion powder to absorb water more slowly. However, the modified CS with high hydrophobicity demonstrated proper physical and oxidative stability, while reducing the redispersibility of powder emulsions.

## 4 CONCLUSION

Glucose consumption and oil production of *Mortierella alpine* CBS 754.68 were tracked during the fermentation time, and the results showed that MgONPs have emerged as a main stimulating factor for acceleration in oil production. The data of the FT‐IR spectrum revealed a successful binding between CS filaments and fatty acids of CA and SA. As a result, modified CS was able to improve the physical and oxidative stability of the emulsions. The rheology tests demonstrated that CS‐stabilized emulsion had a liquid‐like behavior, but modified‐CS‐stabilized emulsion was of a solid‐like behavior. The findings of this study showed that there was a positive correlation between the length of the fatty acid chain attached to chitosan and the emulsifying properties of modified chitosan. In conclusion, the hydrophobic characteristic of chitosan enhanced by longer chain acids was of the best protective effect against oxidation of *Mortierella alpina'* oil and probably performs the same way for the other microbial oil.

## CONFLICT OF INTEREST

The authors declare that they do not have any conflict of interest.

## ETHICAL APPROVAL

This study does not involve any human or animal testing.

## Data Availability

The original data used to support the findings of this study are available within the article.
